# On Neuralgic Affections Following Injuries of Nerves

**Published:** 1864-11

**Authors:** J. Mason Warren

**Affiliations:** Surgeon at the Massachusetts General Hospital


### ON NEURALGIC AFFECTIONS FOLLOWING INJURIES OF NERVES.

By J. MASON WARREN, M.D., Surgeon at the Massachusetts General Hospital.

Injuries of the nerves belong more especially to military surgery, and have therefore, until very lately, been but little studied among us. The information given in the common handbooks is also quite meagre, and eminent authorities differ widely upon important points both of prognosis and treatment. I have thought it worth the while, therefore, to collect the opinions of a few leading surgeons upon this subject, and to compare their teachings with the results of my own experience in a few cases which have recently been under my care.

The immediate effects of the division or injury of a large nerve, are the loss of sensation and of motion, and a diminished power of resisting changes of temperature which would ordinarily cause no inconvenience. Severe pain is also a very common symptom, but is not always observed until after the lapse of a certain time after the receipt of the injury. The loss of sensation and of motion may be either temporary or permanent, as might naturally be expected, but the connection between the precise nature of the injury and the subsequent phenomena has not often been marked out with so much exactness as could be desired. I shall confine myself chiefly to the question of the treatment of neuralgia following injuries of the nerves, commencing by a few quotations from good authorities.

"The best means of mitigating the pain," says Mr. Guthrie (*Commentaries on Surgery*), "independently of the application of warmth, is by the application of stimulants to the whole of the extremity affected, followed by narcotics." The particular applications suggested are tincture of iodine or of cantharides, oil of turpentine, croton oil, liquor ammoniæ, veratria, &c., using them "of such strength as to cause some irritation on the skin, short, however, of producing any serious eruptions." He would follow these stimulant remedies by opium, belladonna, or hyoscyamus applied in the form of an ointment; or by the tinctures of the same narcotics or of aconite applied on linen; he also suggests the employment of aconitia made into an ointment with lard in the proportion of one grain to a drachm.

Dr. Hennen (*Principles of Military Surgery*, Chapter XI., on Injuries of Nerves), states that "mechanical injuries of the nerves are entirely beyond the power of art to relieve effectually, but (that) they are objects of great curiosity, and illustrative of many important symptoms that occur in the course of practise." He mentions a secondary paralysis which "very frequently takes place without any immediate injury of the nerve, as in those cases when a ball has passed so close to a large one, or the plexus from which it proceeds, as to occasion an inflammation and consequent thickening of the neurilemma or investing membrane; or when, in the more distant transit of the ball, the tube formed by its passage swells to an extent sufficient to press on the nerve or plexus." In a celebrated case reported by Dr. Denmark, (*Medico-Chirurgical Transactions*, vol. iv.), in which the patient was tormented by a severe neuralgia, corresponding to the parts supplied by the radial nerve below the elbow, amputation was performed with complete relief of the pain, and on dissection the cause of the trouble was discovered in a bit of lead imbedded in the radial nerve. In a comment on this case, Dr. Hennen approves the course adopted, "although the nature of the lesion was suspected previous to amputation, and indeed almost demonstrated by the symptoms and site of the wound." His objections to any attempt to dissect out the nerve and remove the foreign body, are "the extent to which the thickened and diseased state of the investing membrane of the nerves may reach; the certainty of greatly lessening, and perhaps eventually destroying the motion and sensibility of the parts to which they are distributed, by cutting off the communication with the sensorium; the contracted or distorted state in which the limb generally is; and the possibility of exciting universal and highly dangerous commotion of the system." He further states that while "the total division of a partially wounded nerve is the only operation recognized by modern surgery, the experiment is both hazardous and uncertain; he therefore confines himself to venesection, with emollients to the parts."

An interesting case of traumatic neuralgia, lasting several months, finally recovering without an operation, reported by Mr. Longmore, in Mr. Holmes' *System of Surgery*, closely resembles several of my own cases reported in this paper, and will be cited in connection with them to complete the history of this interesting affection.

In the chapter in *Holmes*' *Surgery* on the Diseases of Nerves, Dr. Brown-Séquard has given an able *resume* of various reflex affections following injuries of the nervous trunks and large branches. For the treatment of all these affections, whether taking the form of epilepsy, tetanus, neuralgia, paralysis, &c., he dwells chiefly upon local measures, and among these he gives the preference to the artificial section of the injured or irritated nerve between the brain or spinal cord and the part of the nerve which is altered. "When there is any chance of a persistence of the irritating cause after the time necessary for the reunion of the parts of the divided nerve," he states that "an excision of an inch or two, which will retard reunion, must be made instead of a simple division." He states also that owing to changes which may take place in the nutrition of the nerve above the part originally affected, it may sometimes be proper to divide the nerve at a point considerably higher than would otherwise be necessary, in some cases operating "even as near the nervous centre as safely possible." Besides these cases requiring division of the nerve, there are others in which "all that is necessary is to gain a few days to allow a wound to heal up." In such a case he recommends "to lay bare the nerve above the wound, and to drop sulphuric ether upon it, which operation, especially if repeated, will render the nerve for many days quite unable to transmit any irritation from the original wound." "Amputation of a limb (he says) should never be resorted to with the view of curing reflex epilepsy, tetanus, &c., unless of course this operation happens to be necessary for another purpose." As the next best local means after neurotomy for combating these affections, he advises the employment of "subcutaneous injections of narcotics, just above the wound, or on the irritated nerve, together with applications of emollient and narcotic lotions or poultices on the wound itself." The dose recommended for subcutaneous injection is half a grain ($\frac{1}{2}$) of morphia or one-sixtieth of a grain of atropia; he also makes the important observation "that in many cases of reflex (nervous) affections, the most powerful narcotics, especially opium, are borne in large doses, without any poisonous effect."

The proposal of the plan of treating nervous affections of this kind by neurotomy involves the whole question of the repair of injured and divided nerves, a subject not very fully discussed in works on surgery, and therefore not very familiar to practical surgeons. I have thought it proper therefore to collect such facts as I could upon the subject both of simple division of nerves and also of division with the excision of a longer or shorter portion of the trunk.

As regards the question of reunion of the two ends of a divided nerve, there is no doubt that such a result often occurs. A sufficient proof of this fact is seen in the restoration of nervous action in the trifacial nerve even after the removal of a portion of one of its larger branches for facial neuralgia,* also in the occasional reproduction of the nerves in the foot of the horse when divided or partially excised to conceal or relieve certain forms of lameness. The same fact is also proved physiologically by the experiments of Cruikshank and Haighton upon the vagus of dogs, and anatomically by Meyer, Swan, Tiedemann, and others, who have actually traced the new nervous

* In two cases where I have removed a portion of the inferior maxillary nerve, amounting to about half an inch, for the relief of tic douloureux, regeneration of the nerve with full restoration of its functions afterwards took place. In the first case, operated on by Dr. J. C. Warren, the disease was relieved for six months, when the pain returned in the same spot beyond the excised portions of nerve. A year after the first operation I trephined the bone and excised a second portion of the nerve with permanent relief. In the second case, which is published in the *Transactions of the Boston Society for Medical Improvement*, June, 1858, the lower jaw was trephined near the angle, and half an inch of the nerve removed for a tic douloureux of eight years' duration, confining the patient for most of that time to her couch. This patient was entirely relieved for about a year, at the end of which time, sensibility had become almost wholly restored to the parts beyond. The violent and persistent neuralgia has not returned, although occasional paroxysms are felt at the same spot as before.

filaments in the cicatricial tissue, uniting the cut ends and filling the void caused by the excision of a portion of several lines (and in one case nearly an inch) in length. Clinical observations bearing upon the same point are recorded by various authors. Mr. Syme, in his *Treatise on the Excision of Diseased Joints** (Case VIII., page 88), gives a remarkable case in which the ulnar nerve was wholly divided at the elbow in the operation of excision of that joint, and in which the functions of the nerve were perfectly restored in the course of a few weeks. A subsequent dissection of the arm less than a year after the operation, revealed the fact that perfect union of the cut ends of the nerve had taken place, and that the nervous filaments could be traced from both ends into the intermediate new tissue and apparently also from one end to the other. In a similar case reported by M. Roux, a portion of the ulnar nerve was actually cut away, but in the course of a year sensation had entirely returned, and when the patient was examined fourteen years after the operation the sensation was as perfect as in the other arm.

A very interesting case of union of the median nerve, after division at the wrist-joint by a circular saw, occurred in the practice of Mr. Stanley, and is admirably reported by Mr. Paget. (*Lectures on Surgical Pathology.* Lecture XII.) In this case sensation began to return within ten days or a fortnight, and was nearly complete in the course of a month. In another similar case, also quoted by Mr. Paget, in which the injury was inflicted with a chaff-cutting machine, the subsequent history was much the same as in the boy treated by Mr. Stanley. These cases are cited by Mr. Paget in illustration of a mode of repair which he calls primary union in contradistinction from secondary union, which takes place by the formation of new connecting substance, and generally requires twelve months for even a partial restoration of the nervous function.

In view of these facts it is important to inquire into the propriety of dividing the nerve as a remedy for traumatic neuralgia. In answer to this question, we may state that if the nerve is simply divided, sensation will probably return before the tissues implicated in the original injury have had time to recover their normal condition, and that, therefore, the operation will afford only very transient relief and may have to be repeated several times. If, on the other hand, a portion of the nerve is excised,

* For the reference to this and the following case I am indebted to Dr. R. M. Hodges, who has also called attention to them in his valuable and interesting paper on Excision of Joints.

the restoration of the nervous function will be very much longer in taking place, but there will also be great danger that the repair will be incomplete, or even that it may fail altogether, and thus entail permanent loss both of sensation and of motion. The deliberate removal of a long section of the nerve with a view to the permanent abolition of its functions can be but very rarely indicated, and then only as a "dernier resort," as the possible alternative of amputation.

The rational treatment of these neuralgic affections seems to me to be based on the fact that their natural tendency is to recovery, if only we can keep the patient comfortable, and thus induce him to wait for this tardy relief. This can only be effected by division of the nerve or by the use, either local or general, of narcotics. The protracted use of opium internally in sufficient quantity to relieve the pain, will almost inevitably exert a most pernicious influence on the health, while mere local applications to the skin seem to have very little effect. The great benefit which has been derived from the use of hypodermic injections of morphia for ordinary neuralgia, naturally suggested the propriety of trying them in this affection, and the success which has attended the experiment has been most gratifying.

The following cases of severe traumatic neuralgia, which have lately occurred in my practice, serve to throw light upon certain points in the pathology and treatment of this painful affection. In all these cases the injury seems to have been to the tissues surrounding a nervous trunk rather than to the nerve itself, and the immediate cause of the painful affection which followed would seem to depend upon the effusion of inflammatory products within the dense fibrous neurilemma, thus entangling the nerve in a mass of cicatrical tissue, perhaps also compressing its fibres. The highly favorable result in Case I. may be readily explained by the well-known law of development of new reparative material, by which it becomes gradually assimilated to the proper tissue of the part in which it is deposited. The dissection made in the course of the operation showed that the nerve was then firmly glued to the surrounding tissues, and its release from these connections was followed by perfect relief of the pain, which, however, returned in a diminished degree as soon as the process of cicatrization had again commenced. The pain was then controlled during six months by the daily use of hypodermic injections of morphia, and at the end of this somewhat protracted treatment the neuralgic affection was found to have disappeared, and the nerve had so far recovered its normal condition as to conduct ordinary sensations in a very satisfactory manner. The second and third cases are equally interesting as showing the powerful effect of the narcotic injection, in the one case in relieving the pain, and in the other actually curing it. The case of Mr. Longmore is also added to show the important part which time plays in the cure of all such cases.

CASE I. *Severe Neuralgic Affection following a Gunshot Injury of the Median Nerve.*—In the second battle of Bull Run, Lieut. A., of a N. H. regiment, was struck by a ball on the outside of the middle of the arm. The ball passed obliquely through, traversing the biceps muscle and coming out on the inside. For two or three days he was exposed to the weather, lying under the piazza of a house, having but little food, and with his hand constantly wet with the rain which was falling. The hand was benumbed, but he suffered somewhat with a sensation of heat in it, which was partially relieved by keeping it exposed to the wet. There was no pain in the wound itself. Shortly after he was removed to Washington, where he first experienced very severe pain in the whole hand, but more particularly in the part of it supplied by the median nerve. I saw him about a fortnight after the receipt of the injury. He was then in constant and severe pain in the hand, so much so as to require to be kept more or less under the influence of morphia, which he was taking to the amount of a grain a day. On examining the point at which the wound was received, a puckered eschar was seen with an induration extending deeply into the belly of the biceps muscle to which the skin was adherent. The situation occupied by the vessels and nerves on the inside of the biceps was also enveloped in a mass of indurated tissue. The first idea suggested by this state of things was to cut down upon the nerve, to divide it. It seemed, however, possible, by the gradual change going on in the tissues, that a healthy action might ultimately be set up, and at the same time, the indurated tissue surrounding and compressing the nerve might be absorbed, finally relieving the nerve from pressure. The question was whether the sufferings of the patient could be sufficiently mitigated by artificial means to allow of the adoption of a temporizing course. He was advised to place the limb perfectly at rest, wear it inside of his clothes, next the body, and to have a sleeve made of sheet India rubber to envelop the lower part of the arm, which covering was to be removed from time to time, the arm exposed to the air and washed with soap and water; he was directed to discontinue the use of the rubber sleeve if much irritation was set up in the skin, and to envelop the arm in flannel instead, which he had at all times found necessary owing to the great reduction of temperature. He went home to New Hampshire, and followed this plan for three or four weeks. At the end of that period he came to me again with the desire of having the nerve divided, as his suffering had become so intolerable, in spite of the use of opiates, as entirely to deprive him of rest. Before resorting to an operation on the nerve I determined to try the effect of subcutaneous injections of morphia. Half a grain of sulphate of morphia, in solution, was injected deep under the skin of the forearm twice a day. He was at once placed in a state of comparative ease, and the evening injection gave him a good night's rest, such as he had not enjoyed for many weeks. This plan was followed up for a month with equally good effects; his digestion was not in the least affected by the use of the morphia, and he gained considerably in flesh. If, however, the dose was omitted, the pain became as bad as ever. It was therefore decided to perform an operation. An incision of three inches in length was made over the inner edge of the biceps, and the integument dissected on both sides separating the cicatrices, caused by the entrance and exit of the ball from the subjacent tissues. The indurated mass which surrounded the vessels and nerves was now cut into, and the median nerve being discovered, where it entered was gradually laid bare and dessected out so that it lay perfectly loose in the wound for an inch and a half or two inches of its length. It was thought best not to divide the nerve, but to await the result of the healing of the wound. The edges of the wound were loosely approximated, and water dressings applied. For some days the pain was entirely relieved, although from the effect of the habitual use of morphia, a small dose was required to promote sleep. As the wound began to heal, however, the pain returned, but was much less severe than before. Desiring now to return home, one of his family was instructed in the use of the subcutaneous injection of morphia. About two months afterwards he called on me, and again (March 20, 1863), four months after the operation. He was then in a state of perfect health, and had gained much flesh, but complained still of neuralgic pain in the head, requiring the employment of the narcotic injection, whether from habit or not seemed to be a question. The arm, hand, and fingers had begun to acquire some motion. In regard to the local effect of the injections it my be said, that although they had been used twice a day for five months, he had never suffered from any irritation at the point of puncture except in one instance in the case of a freshly prepared solusion of sulphate of morphia, the use of which was followed by the production of a large red blotch whenever it was injected. On substituting a solution of acetate of morphia no farther trouble of this nature was experienced.* The patient had, therefore, had nearly three hundred injections of morphia, more or less, and with the above exception no traces remained of its protracted use.

*Oct.* 26, 1863. I have just seen this patient, and find that he has recovered his health and enjoys complete immunity from pain. The hypodermic injections were continued until the month of July, or about nine months from the receipt of the injury. He then, by a great effort, suddenly discontinued them, and has not used them since. The neuralgic affection, except during extreme changes of the weather, has left him. The forearm has recovered its natural sensibility; he has the power of complete flexion of the elbow, and of partial rotation of the forearm, while the fingers, which were formerly held in a state of extension, can now be approximated to the thumb, so as to make the hand useful for most of the ordinary purposes of life. This motion is continually improving.

CASE II. *Gunshot Wound of the Thigh implicating the Sciatic Nerve.*—I have lately had under my care, in the hospital, a soldier, who, two months before, was shot in the thigh, and was taken prisoner. The ball traversed the thigh from side to side, and, probably, injured the sciatic nerve, in whose immediate neighborhood it must have passed. He suffered no inconvenience in the site of the wound, but shortly afterwards a severe neuralgic pain commenced in the sole of the foot, accompanied by a sensation of heat and great tenderness of the part, and entirely incapacitating him for locomotion. Opiates in the usual form gave him but little relief, and the only alleviation of his sufferings while in prison at Richmond was obtained by keeping the leg constantly plunged in a pail of cold water. I immediately ordered the subcutaneous injection of a quarter of a grain of morphia daily into the leg, and gradually increased the dose to a grain a day. By this treatment the pain was completely held in check, rendering his days and nights comfortable. The full effect of each dose was obtained in from five to ten minutes after injecting it.

In the course of this case I experimented as to the effect of the injection, when made at a distant part of the body, as com-

* This accident is probably to be explained by the common practice of adding free sulphuric acid to promote the solubility of certain specimens of sulphate of morphia. The acetate is very soluble in water.

pared with its effect when applied in the immediate vicinity of the affected nerve. I found that when the injection was made in the opposite limb to that affected, the relief was as prompt and as complete as when made directly over the course of the nerve; and this occurred repeatedly, in every instance in which it was tried. This is a point of very considerable importance, inasmuch as it is often very inconvenient to make the injection in the exact situation of the affected nerve, as has been strongly insisted on by several writers upon this subject.

CASE III. *Injury of the Ulnar and Musculo-Spiral Nerves, from a Bullet.*—Captain C——, who had already been twice wounded in the thigh and leg in the battles of Winchester and Fredericksburg, was hit at the battle of Gettysburg by a ball just over the median nerve. It passed in a spiral direction around the bone, and came out half way down the limb below on the other side. The hand and forearm were at once partially paralyzed, and in a day or two very severe neuralgic pains commenced, principally in that part of the hand supplied by the ulnar nerve. When I first saw him, about a week after the injury, the arm was much swollen, and the wounds, which had still on them the cold water dressing, were in an irritable state, and there was no appearance of suppuration. The water dressings were replaced by a large warm poultice, and on a free suppuration being established the extreme pain in the arm and hand was much relieved. The pain, however, still continued to recur at intervals, and the paroxysms coming on at night were very severe. Finally the hypodermic injection of morphia was tried, and a single dose of $\frac{1}{2}$ grain afforded entire relief for the time being, and in fact distroyed the habit so that the paroxysms did not recur. The hand and arm, however, for a long time afterwards were very uncomfortable on account of the excessive heat of the parts, which was only relieved by the constant use of cold water, and it was not until after several months that the normal sensibility began to return, and this symptom to disappear. Seen again at the end of five months, he was free from neuralgic pain, had some use of his hand, and the elbow has become flexible after employing forcible extension to overcome stiffness produced partly by inaction and partly by the contraction of the injured muscles. The movement of rotation of the forearm had not yet been recovered by the patient, although they could be easily made by a second person, the nervous power being still defficient.

Case recorded by Mr. Longmore (Holmes' *System of Surgery*, vol. ii. p. 88).

"A soldier of the 37th Regt. was wounded at Azimghur on the 27th of March, 1858, by a musket ball through the right side of the neck. It entered just below the horizontal ramus of the jaw, and made its exit behind, over the scapula. About three pints of blood escaped, supposed to be from the external jugular vein. The wound healed favorably, but he lost the use of his right arm, at first completely, and afterwards partially, for three months. At the expiration of that period the power of the arm was restored, but he was invalided home on account of severe pain in the back of the neck, 'resembling toothache,' which all treatment failed to relieve. The pain spontaneously and gradually ceased; there is still some loss of of substance of the trapezius muscle of the right side of the neck, and of the right as compared with the other arm, with occasional numbness, when the man is in heavy marching order; but in all other respects he is well, and is at his regular duty.—*American Journal of the Medical Sciences.*

